# Transcutaneous Vaccination with Conjugate Typhoid Vaccine Vi-DT Induces Systemic, Mucosal, and Memory Anti-Polysaccharide Responses

**DOI:** 10.4269/ajtmh.19-0798

**Published:** 2020-07-27

**Authors:** Md Saruar Bhuiyan, Anuj Kalsy, Mohammad Arifuzzaman, Richelle C. Charles, Jason B. Harris, Stephen B. Calderwood, Firdausi Qadri, Edward T. Ryan

**Affiliations:** 1 International Center for Diarrheal Disease Research, Bangladesh (icddr,b), Dhaka, Bangladesh;; 2 Division of Infectious Diseases, Massachusetts General Hospital, Boston, Massachusetts;; 3 Department of Medicine, Harvard Medical School, Boston, Massachusetts;; 4 Department of Pediatrics, Harvard Medical School, Boston, Massachusetts;; 5 Department of Microbiology, Harvard Medical School, Boston, Massachusetts;; 6 Department of Immunology and Infectious Disease, Harvard School of Public Health, Boston, Massachusetts;

## Abstract

Transcutaneous vaccination can induce both mucosal and systemic immune responses. However, there are few data on anti-polysaccharide responses following transcutaneous vaccination of polysaccharides, despite the role that anti-polysaccharide responses play in protecting against intestinal mucosal and respiratory pathogens. Whether transcutaneous vaccination with a conjugate polysaccharide vaccine would be able to induce memory responses is also unknown. To address this, we transcutaneously vaccinated mice with virulence antigen (Vi) polysaccharide of *Salmonella enterica* serovar Typhi (the cause of typhoid fever), either in unconjugated or conjugated form (the latter as a Vi-DT conjugate). We also assessed the ability of the immunoadjuvant cholera toxin to impact responses following vaccination. We found that presenting Vi in a conjugate versus nonconjugate form transcutaneously resulted in comparable serum IgG responses but higher serum and lamina propria lymphocyte IgA anti-Vi responses, as well as increased IgG memory responses. The addition of immunoadjuvant did not further increase these responses; however, it boosted fecal IgA and serum IgG anti-Vi responses. Our results suggest that transcutaneous vaccination of a conjugate vaccine can induce systemic as well as enhanced mucosal and memory B-cell anti-polysaccharide responses.

## INTRODUCTION

Transcutaneous immunization (TCI) is a needle-free patch vaccine delivery method that has been shown to be an effective way to induce both mucosal and systemic immune responses and may be an attractive option for non-parenteral immunization, including in resource-limited areas of the world.^1–4^ Immune responses against polysaccharides are associated with protection against a number of bacterial pathogens; however, little is known about immune responses to polysaccharide antigens applied transcutaneously. To our knowledge, there has been no previous direct comparison of immune responses following transcutaneous presentation of the same polysaccharide as a free antigen versus being presented in a conjugate form. We took advantage of the availability of the virulence antigen (Vi) polysaccharide of *Salmonella enterica* serotype Typhi in both conjugate and nonconjugate forms to assess such a comparison.

The model that we used (Vi versus Vi-DT) is based on vaccines against typhoid fever. Typhoid fever remains a global public health problem in the developing world, with an estimated 14.3 million cases and 116,815 deaths each year.^5,6^ Typhoid is caused by infection with *S. enterica* serovar Typhi, a human-restricted pathogen that enters via the gastrointestinal tract and crosses the intestinal mucosal border before disseminating in the blood and reticuloendothelial system.^7^ The appearance of *S.* Typhi that are multiply resistant to commonly used antibiotics has restricted the ability to treat patients with typhoid fever with previously used therapies, now requiring treatment with later-generation antibiotics not always available or affordable in resource-limited settings. Therefore, a major strategy in mitigating the burden of typhoid fever globally has been through vaccination.^8^

A number of typhoid vaccines are currently available.^9^ An attenuated strain of *S.* Typhi Ty21a can be given orally which provides excellent protection for 3 or more years. However, this vaccine requires cold storage and three or four doses to be effective. The Vi polysaccharide is present in the capsule of strains of *S.* Typhi, and Vi-specific antibody responses correlate with protection against typhoid.^10^ Vi polysaccharide administered parenterally as a single-dose vaccine provides up to 2 years of protection against typhoid fever. Newer versions of the Vi vaccine have been developed that have conjugated the Vi polysaccharide to protein carriers, such as diphtheria toxoid (DT), and the physical, chemical, and immunologic properties of Vi-DT conjugate have been well characterized.^11^ Recent studies suggest that conjugation of Vi to DT improves immunogenicity, especially in young children,^5,12^ and results in more prolonged immune responses and longer term protection following parenteral vaccination through the induction of T-cell–dependent memory B-cell responses to the Vi polysaccharide.^5,12^

In this study, we evaluated systemic, mucosal, and memory B-cell immune responses to Vi and Vi-DT conjugate vaccines when administered transcutaneously in a mouse model, with and without cholera toxin (CT) given at the same transcutaneous site as an adjuvant.

## MATERIALS AND METHODS

### Mice.

Three- to 5-week-old female Swiss Webster mice were purchased from Taconic Farms, Hudson, NY. All the animals were maintained in the Center for Comparative Medicine animal facility at the Massachusetts General Hospital under standard laboratory conditions and were given sterile water and routine animal food throughout. Mice were handled and disposed of according to the guidelines of the Institutional Animal Care and Use Committee. This work was approved by the Massachusetts General Hospital Subcommittee on Research Animal Care. The work adheres to the U.S. Department of Agriculture Animal Welfare Act, PHS Policy on Humane Care and Use of Laboratory Animals, and the *Institute for Laboratory Animal Research* *Guide for the Care and Use of Laboratory Animals*.

### Antigens for immunization and laboratory assays.

Purified Vi polysaccharide and DT-conjugated Vi polysaccharide (Vi-DT) (both gifts from the International Vaccine Institute, IVI, in South Korea), and CT (CT; List Biological Laboratories Inc., Campbell, CA) were used for immunizations. Each mouse was transcutaneously immunized with 25 µg of Vi, 25 µg of Vi in Vi-DT conjugate, or 10 µg of CT alone or as an adjuvant with Vi or Vi-DT. Typhim Vi (Sanofi Aventis, Bridgewater, NJ), CT, and keyhole limpet hemocyanin (KLH; Sigma Chemical Co., St. Louis, MO) were used for detection of immunologic responses.

### Immunization regimen and schedule.

Five different groups of mice (*N* = 10–15 each) were immunized transcutaneously as previously described^13^ with CT, Vi, Vi conjugated with DT (Vi-DT), or Vi or Vi-DT with CT as an adjuvant.

**Table:** 

Group	Antigens	Amount of antigen (µg)	Number of mice
Group 1	CT	10	15
Group 2	Vi	25	11
Group 3	Vi + CT	25 of Vi and 10 CT	12
Group 4	Vi-DT	25 of Vi	10
Group 5	Vi-DT + CT	25 of Vi and 10 of CT	15

To judge kinetics of responses, mice were immunized on days 0, 14, 28, 42, and 72. We obtained blood on days 0 (before immunization), 28, 56, and 87; stool on day 87; and euthanized mice on day 87 to obtain lamina propria lymphocytes (LPLs) and spleen memory B cells as previously described.^13^

### Detection of Vi and CT-specific antibody responses in serum.

We measured serum IgG and IgA ELISA antibody responses to Vi and CT, as described before.^14,15^ We coated 96-well plates with either Vi (200 ng/well) or CT (100 ng/well) overnight at room temperature. Sera (diluted 1:25 in 0.1% bovine serum albumin (BSA) in phosphate buffered saline [PBS]-Tween) were added at 100 µL/well and incubated for 90 minutes at 37°C. Anti-mouse IgA or IgG antibody (diluted 1:1,000 in 0.1% BSA in PBS-Tween) (Southern Biotech, Birmingham, AL) was added to the plate and incubated for 90 minutes at 37°C. The plates were washed (three times with PBS-Tween and once with PBS) and developed with colored substrate, ABTS/H_2_O_2_ (Sigma-Aldrich, St. Louis, MO). We measured absorbance values at 405 nm and used kinetic readings (milli absorbance/second) in SoftMax Pro software (version 5.3, Sunnyvale, CA).

### Detection of Vi and CT-specific antibody responses in stool.

We prepared fecal extracts by placing one stool pellet in 1 mL of a 3:1 mixture of PBS–0.1 M ethylenediaminetetraacetic acid (EDTA) containing soybean trypsin inhibitor (Type II-S; Sigma) at a concentration of 0.1 mg/mL and vortexed until the pellet was broken. We centrifuged the mixture twice, added 20 µL of 100 mM phenylmethylsulfonyl fluoride (Sigma) to each 1 mL of the final recovered supernatant, and stored samples at 70°C for later analysis.

We measured fecal IgA antibody responses by ELISA. We coated 96-well plates with either Vi (200 ng/well) or CT (100 ng/well) overnight at room temperature. Sera (diluted 1:25 in 0.1% BSA in PBS-Tween) were added at 100 µL/well and incubated for 90 minutes at 37°C. Anti-mouse IgA (diluted 1:1,000 in 0.1% BSA in PBS-Tween) (Southern Biotech) was added to the plate and incubated for 90 minutes at 37°C. The plates were washed (three times with PBS-Tween and once with PBS) and developed with colored substrate, ABTS/H_2_O_2_ (Sigma-Aldrich). We measured absorbance values at 405 nm and used kinetic readings (milli absorbance/second) in SoftMax Pro software (version 5.3) to determine the ELISA values.

### Isolation of LPLs.

We euthanized mice on day 87 and collected the intestines of mice into Roswell Park Memorial Institute (RPMI) complete media, and kept these at 4°C until processing. Peyer’s patches were removed from the intestine under a magnifying glass. Stool was removed manually from the intestine and the tissue washed once with HBSS media supplemented with 5% fetal calf serum (FCS) (Hyclone, Logan, UT) and 1% gentamicin. The intestine was cut into small pieces with fine scissors, washed three times with the aforementioned media, and incubated with 0.5 M EDTA for 20 minutes at 37°C. The intestinal pieces were allowed to settle in a Falcon tube, and the supernatant was aspirated. The intestine was washed twice with RPMI supplemented with 5% FCS. Collagenase (Sigma-Aldrich, San Francisco, CA) was added to the intestinal pieces and incubated for 30–35 minutes at 37°C. After settling of the residual tissue, the supernatant containing the cell suspension was collected and stored at 4°C. The intestinal pieces were treated twice more with collagenase treatment, and after each treatment, the supernatants containing the cell suspensions were collected and pooled. Lamina propria lymphocytes were isolated from the cell suspension by density gradient centrifugation through Ficoll-Isopaque as previously described.^16^ Lamina propria lymphocytes were resuspended in PBS, and the number of lymphocytes recovered was counted in a hemocytometer.

### Isolation of spleen lymphocytes.

Spleens were harvested from the euthanized mice on day 87. Cells were recovered by teasing the tissue apart using mechanical agitation between pre-cleaned frosted microscope glass slides (Fisher Scientific, Pittsburgh, PA), and cells were suspended in RPMI complete media containing L-glutamine (Invitrogen, Carlsbad, CA) supplemented with heat-inactivated 10% FCS, 3-mercaptoethanol (Sigma) and antibiotics (1% penicillin and streptomycin). Erythrocytes were removed from the splenic cell suspension by osmotic shock using red blood cell lysing buffer (Sigma Chemical Co) as described elsewhere.^17^ The number of cells recovered was counted in a hemocytometer as mentioned previously and resuspended in RPMI complete media at a concentration of 5 × 10^5^ cells/mL.

### Antibody secreting cell (ASC) assay.

Antibody secreting cells were measured in recovered LPLs to detect total and antigen-specific IgG and IgA responses against Vi, CT, and KLH using an ELISpot assay as described previously.^18^

In brief, nitrocellulose-bottomed 96-well MultiScreen HA filtration plates (Millipore Corporation, Bedford, MA) were coated with antigens of interest (anti-mouse IgA [1:500 dilution] and IgG [1:500 dilution]) to count all ASCs, and the specific antigens Vi (200 ng/well), CT (100 ng/well), and KLH (100 ng/well as a negative control) overnight at 4°C. Plates were washed once using PBS containing 0.1% Tween 20 (PBS-T) and two times with PBS. Blocking media consisting of RPMI complete media supplemented with 10% fetal bovine serum was added to each well for 2 hours at room temperature. This blocking media was removed, the plates were washed again three times with PBS, and the LPL cell suspensions were added to the wells in a total volume of 100 µL each.

Plates were then incubated at 37°C, 100% humidity, and 6% CO_2_ conditions for 5–7 hours. Plates were emptied by flicking and washed four times using PBS-T (0.05%) and two times using PBS. Then, 100 µL of biotinylated anti-mouse IgG or IgA (Southern Biotech) in PBS-T (0.05%)-FCS (1%) was applied to all wells, and plates were incubated at 4°C overnight. Following incubation, plates were washed four times with PBS-T. And then, 100 µL of horseradish peroxidase (HRP)–conjugated avidin D (Vector Laboratories, Burlingame, CA) was added at 5 µg/mL in PBS-T (0.05%)-l% FCS and incubated at 37°C for one hour. Following incubation, plates were washed three times with PBS-T (0.05%) and three times with PBS. Then, 100 µL of freshly prepared HRP–H_2_O_2_ chromogen substrate was added for developing the plates. The substrate was prepared using AEC (3-amino-9-ethyl carbazole) (MP Biochemicals, Solon, OH) at a concentration of 10 mg/mL in dimethylformamide) in 0.1 M sodium acetate buffer (pH = 4.8); the solution was then filtered using a 0.2-µm membrane. Just before use, 100 µL of 3% H_2_O_2_ (Fisher Scientific) was added. Round red colored spots appeared in about 5 minutes. The reaction was stopped by rinsing plates thoroughly with tap water. Plates were allowed to dry, and spots were analyzed using a light microscope. The number of antigen-specific ASCs counted was expressed as a percentage of the total number of ASCs of that same isotype. Responder frequency was determined as the percentage of mice that had a measurable ASC number recorded.

### Memory B-cell assay.

Splenocytes were diluted to concentrations of 1 × 10^5^ and 1 × 10^4^ per ml in 96-well flat bottom plates (Nunc, Nalgene International, Rochester, NY). Plates were supplemented with 8 × 10^5^ irradiated (1,200 rads) syngeneic spleen cell feeders per well derived from naive mice.^16^ Concanavalin A (ConA)–supplemented master media mix (20 ng/mL PMA, 2.5 μg/mL ConA, 1 mg/mL R595 LPS, Sigma) was added to the stimulated wells and RPMI alone to the unstimulated wells, totaling to 200 µL volume per well.^16^ Cells were cultured for 6 days at 37°C, 5% CO_2_, and 100% humidity. After culturing, each cell suspension was transferred to 96-well U-bottom plates (Nunc, Nalgene International) and centrifuged at 1,000 rpm in a tabletop centrifuge (Fisher Scientific). Cells were washed with 200 μL of RPMI complete media. Each cell pellet was resuspended in 100 μL of RPMI complete media and transferred to nitrocellulose-bottomed 96-well plates (Millipore) previously coated with anti-mouse IgG (Southern Biotech), Vi, CT, or KLH and processed as described earlier for the ASC assay. We excluded any samples that had spots in the unstimulated well for antigen-specific responses, or more than two spots in the KLH well after stimulation. We used stimulation of approximately 150% over unstimulated wells for the total number of memory B cells to consider the results to be usable for analysis.

### Statistical analyses.

Statistical analyses were performed using GraphPad prism software version 6 (GraphPad Software, Inc., La Jolla, CA). Statistical evaluation of differences between study days was performed using the Wilcoxon signed-rank test. Two-sided *P* values ≤ 0.05 were considered significant. Figures were generated in GraphPad prism.

## RESULTS

### Vi-specific antibody responses in serum.

**Figure 1.**
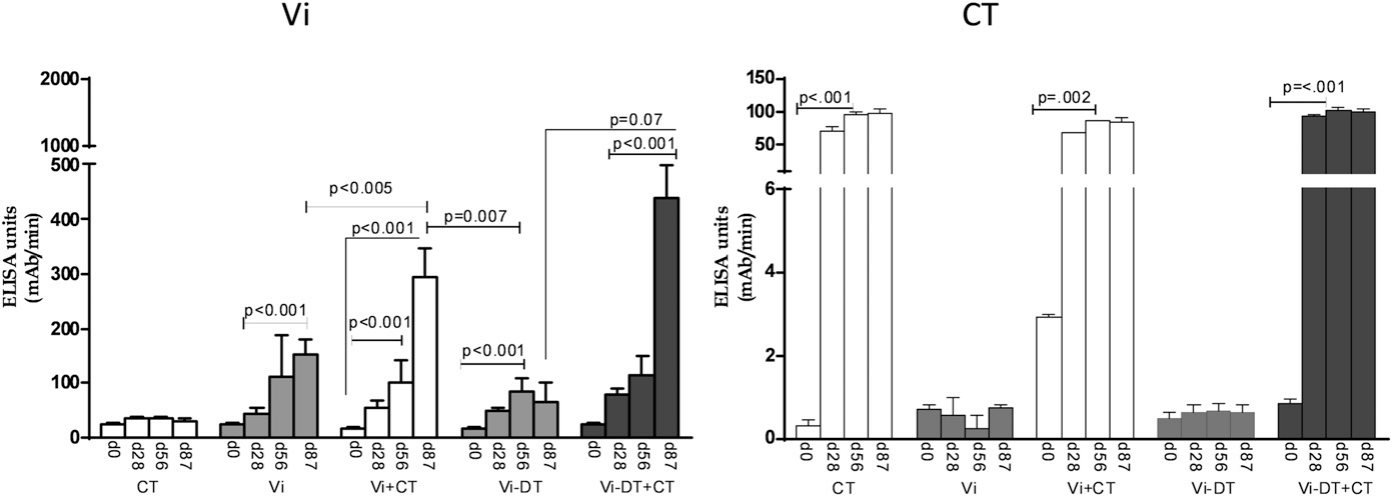
Serum IgG antibody responses against virulence antigen (Vi) and cholera toxin (CT) in immunized mice. Vi and CT-specific IgG responses were measured on days 0, 28, 56, and 87 in sera of mice immunized with different antigens. Each column indicates mean with standard error of the mean. Statistically significant differences relative to the compared group are indicated.

**Figure 2.**
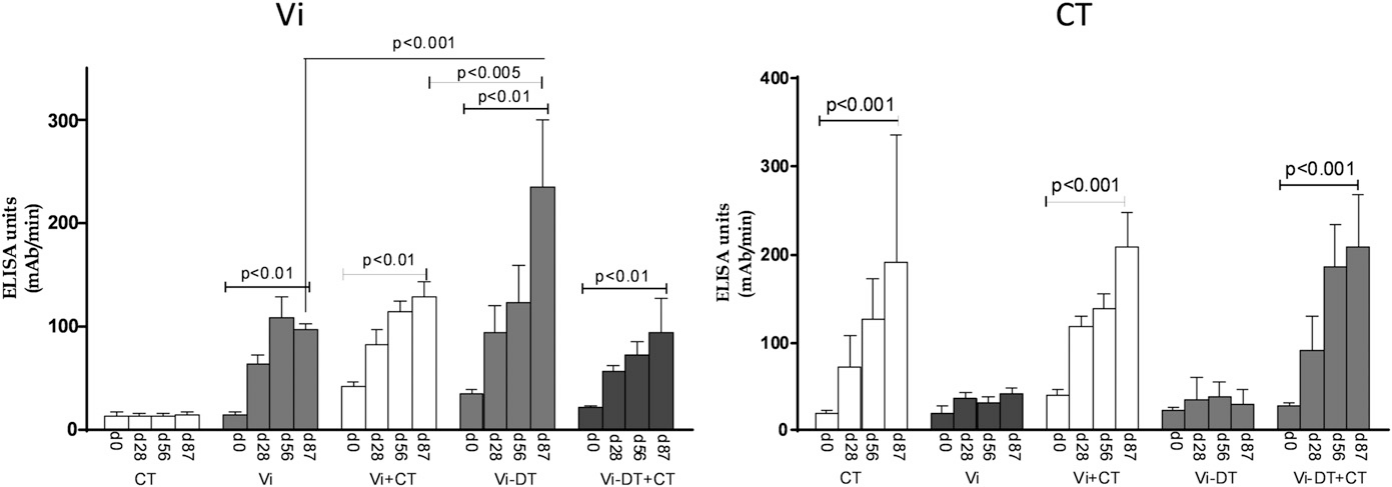
Serum IgA antibody responses against virulence antigen (Vi) and cholera toxin (CT) in immunized mice. Vi and CT-specific IgA responses were measured on days 0, 28, 56, and 87 in sera of mice immunized with different antigens. Each column indicates mean with standard error of the mean. Statistically significant differences relative to the compared group are indicated.

Transcutaneous immunization with Vi polysaccharide induced both IgG (Figure 1) and IgA (Figure 2) antibody responses in serum against Vi antigen. Immunization with Vi-DT also induced significant serum IgG and IgA responses (Figures 1 and 2), and the serum IgA response to Vi was significantly higher following vaccination with Vi-DT than unconjugated Vi. Addition of CT to Vi or Vi-DT induced significantly higher IgG antibody responses to the Vi antigen (Figure 1), but the addition of CT as adjuvant did not have a significant effect on IgA responses (Figure 2).

### Cholera toxin-specific antibody responses in serum.

Mice immunized with CT with or without Vi or Vi-DT developed significant IgG and IgA antibody responses in serum (Figures 1 and 2). There were no significant CT responses in mice immunized with Vi or Vi-DT transcutaneously without CT.

### Vi-specific antibody responses in fecal extracts.

**Figure 3.**
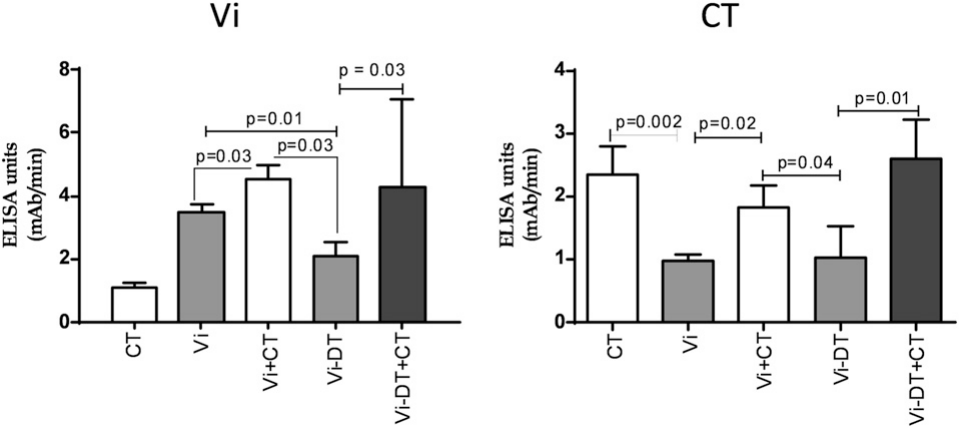
IgA responses against virulence antigen (Vi) and cholera toxin (CT) in fecal extracts of immunized mice on day 87. IgA antibody responses to Vi and CT in fecal extracts of immunized mice at day 87. Each column indicates mean with standard error of the mean. Statistically significant differences relative to the compared group are indicated.

There was a significant anti-Vi IgA response in fecal extracts of mice receiving Vi transcutaneously (Figure 3). Addition of CT as adjuvant induced significantly higher anti-Vi IgA responses in fecal extracts from mice receiving either Vi or Vi-DT transcutaneously (Figure 3).

### Cholera toxin-specific responses in fecal extract.

Similar to serum antibody responses, TCI with CT with or without Vi or Vi-DT induced significant IgA responses in fecal extracts of immunized mice (Figure 3).

### Antibody-secreting cell responses in LPLs recovered from intestines of immunized mice.

**Figure 4.**
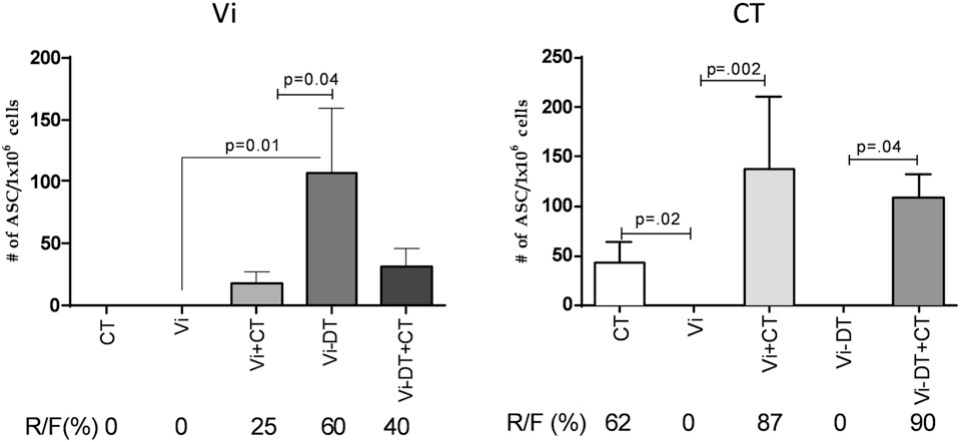
Anti-virulence antigen (Vi) and anti-cholera toxin (CT) IgA ASC responses in LPLs recovered from immunized mice on day 87. Antigen-specific IgA antibody-secreting cell responses to Vi and CT in LPLs recovered from immunized mice on day 87. Each column indicates mean with standard error of the mean. Statistically significant differences relative to the compared group are indicated.

We investigated mucosal immune response in the gut in recovered LPLs following TCI. Although Vi TCI did not produce any detectable IgA Vi-specific ASCs in recovered LPLs, addition of CT to Vi TCI did produce a small, although not significant, increase in Vi-specific IgA ASCs (Figure 4). Transcutaneous immunization with the Vi-DT conjugate produced significantly more Vi-specific IgA ASCs in recovered LPLs than Vi alone (Figure 4). The addition of CT to Vi-DT did not augment the number of Vi-specific IgA ASCs in LPLs. The CT-specific IgA ASCs in recovered LPLs were seen only in mice that received CT transcutaneously with or without Vi or Vi-DT. (Figure 4).

### Vi-specific IgG memory B-cell responses in spleen.

**Figure 5.**
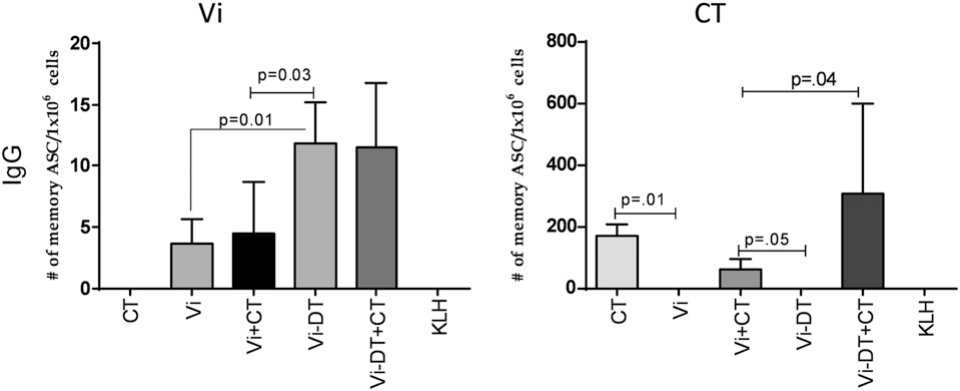
IgG memory B-cell virulence antigen (Vi) and cholera toxin (CT)-specific responses in immunized mice on day 87. Mean antigen-specific IgG memory B-cell responses to Vi and CT in spleens of immunized mice on day 87, expressed as the percentage of total memory B cells of that isotype. Error bars represent standard errors of the mean. Statistically significant differences relative to the compared group are indicated.

We assessed the development of IgG memory B cells in spleens from immunized mice to determine the ability of transcutaneous Vi or Vi-DT conjugate vaccine to induce memory B-cell responses (Figure 5). We observed low levels of Vi-specific IgG memory B cells following immunization with Vi alone, and these did not increase further with addition of adjuvant CT. Transcutaneous immunization with Vi-DT induced significantly higher IgG memory B-cell responses than Vi itself, but these were not increased further by addition of adjuvant CT. IgG memory B-cell responses to CT were seen only in mice that received transcutaneous CT with or without Vi or Vi-DT (Figure 5).

## DISCUSSION

Little is known about immune processing of polysaccharides when delivered transcutaneously, especially if the sugar is presented in a free versus conjugate form. Mawas et al.^19^ previously vaccinated rats transcutaneously with *Haemophilus influenzae* type b polysaccharide conjugated to CRM197, administered with or without immunoadjuvants, and found that anti-polysaccharide IgG responses developed. However, in that experiment, there was no comparison made to vaccination with polysaccharide alone nor were IgA or mucosal responses reported. Rollenhagen et al.^4^ transcutaneously vaccinated mice with a synthetic short neoglycoconjugate cholera vaccine with or without adjuvant, comparing responses with those induced following subcutaneous vaccination. They found prominent IgG responses, with both transcutaneous and parenteral vaccination, but no appreciable mucosal responses. They also did not compare responses with those induced following vaccination with polysaccharide alone. Tarique et al.^15^ found that orally priming mice with oral cholera vaccine before transcutaneous vaccination with a cholera conjugate vaccine not only induced IgG responses but also induced mucosal IgA and IgM responses. Once again, there was no comparison with responses following transcutaneous vaccination with polysaccharide alone. Memory responses were also not evaluated in any of these studies.

In our current analysis, we took advantage of having access to both purified Vi vaccine and Vi-DT conjugate vaccine. Our results corroborate that TCI with conjugate vaccine not only can induce prominent systemic anti-polysaccharide responses but also can induce mucosal responses. Presenting the Vi antigen in a conjugate form increased systemic IgA responses and IgA responses in LPLs in mucosal tissue. Presentation of polysaccharide in a conjugate form also resulted in prominent IgG memory B-cell responses that were not seen following presentation of sugar alone. Our results are similar to those reported by Sayeed et al.^16^ who found that intradermal vaccination of a cholera conjugate vaccine induced not only systemic IgG and IgA responses but also an IgA response in LPLs and memory anti-polysaccharide responses. Our current analysis of transcutaneous vaccination suggests that a conjugate vaccine can transit the epidermis and be immunologically processed, presumably by intradermal dendritic cells.^20,21^ The induction in our study of both mucosal and systemic immune responses following TCI of a conjugate vaccine corroborates the previously described interaction between the dermal and mucosal immune systems.^4,15,19–21^ The induction of memory responses following TCI of a conjugate vaccine in our study suggests involvement of T cells in antibody maturation, as would be expected when a polysaccharide is presented in a conjugate form.

Previous studies suggested improved immunogenicity to transcutaneously applied antigens including polysaccharides, when administered with adjuvants including adenosine diphosphate (ADP)-ribosylating agents.^15,19,22^ In our current analysis, the addition of CT did indeed increase systemic IgG responses but not responses in LPLs or memory responses. Interestingly, the addition of CT to Vi-DT resulted in a trend toward reduction of serum IgA anti-Vi response compared with Vi-DT alone. A similar trend in anti-Vi IgA ASC responses was also noted. Similar trends were not noted in anti-protein responses. Whether these nonstatistically significant trends suggest that ADP-ribosylating immunoadjuvants might blunt mucosal polysaccharide-specific IgA responses following transcutaneous application of polysaccharide in a conjugate form is currently uncertain. Whether such an effect would be evidenced with other ADP-ribosylating adjuvants such as modified version of heat labile enterotoxin (mLT and dmLT) is also uncertain.^23^ In this study, we used CT because of its availability.

One limitation of our study is that we did not perform a direct comparison with immune responses following parenteral vaccination; however, the central purpose of our analysis was to compare immune responses to a polysaccharide antigen when it is presented to the dermal immune system as a conjugated versus nonconjugated form. Our results suggest that transcutaneous vaccination with conjugate Vi-DT vaccine can induce both systemic and mucosal responses, as well as IgG memory responses against a polysaccharide antigen. Because typhoid fever is a systemic infection, whether such responses would improve vaccine-protective efficacy compared with parenteral vaccination against Vi is uncertain. However, our results suggest the feasibility of an alternate vaccination strategy for polysaccharide-based conjugate vaccines that might be particularly attractive as a way to induce high-level mucosal, systemic, and memory anti-polysaccharide immune responses for noninvasive mucosal pathogens.
